# A prognostic model incorporating the albumin-corrected anion gap in patients with aneurysmal subarachnoid hemorrhage

**DOI:** 10.3389/fneur.2024.1361888

**Published:** 2024-06-19

**Authors:** Ruoran Wang, Juan Rong, Jianguo Xu, Min He

**Affiliations:** ^1^Department of Neurosurgery, West China Hospital, Sichuan University, Chengdu, Sichuan, China; ^2^West China Centre of Excellence for Pancreatitis, Institute of Integrated Traditional Chinese and Western Medicine, West China Hospital, Sichuan University, Chengdu, Sichuan, China; ^3^Department of Critical Care Medicine, West China Hospital, Sichuan University, Chengdu, Sichuan, China

**Keywords:** serum anion gap, albumin, aneurysmal subarachnoid hemorrhage, prognosis, marker

## Abstract

**Background:**

Aneurysmal subarachnoid hemorrhage (aSAH) patients typically have poor prognoses. The anion gap (AG) has been proven to correlate with mortality in various critically ill patients. However, hypoalbuminemia can lead to underestimations of the true anion gap levels. This study was conducted to verify the prognostic value of single AG and albumin-corrected anion gap (ACAG) among aSAH patients.

**Methods:**

Significant factors in the univariate logistic regression analysis were included in the multivariate logistic regression analysis to explore the risk factors for mortality in aSAH patients and to confirm the independent relationship between ACAG and mortality. The restricted cubic spline (RCS) was used to visually show the relationship between ACAG level and mortality risk of aSAH patients. The predictive model for mortality was developed by incorporating significant factors into the multivariate logistic regression analysis. The prognostic value of ACAG and the developed model was evaluated by calculating the area under the receiver operating characteristics curve (AUC).

**Results:**

Among 710 aSAH patients, a 30-day mortality was observed in 20.3% of the cases. A positive relationship was demonstrated between the ACAG level and mortality in aSAH patients using the RCS curve. The multivariate logistic regression analysis helped discover that only six factors were finally and independently related to mortality of aSAH patients after adjusting for confounding effects, including the Hunt–Hess scale score (*p* = 0.006), surgical options (*p* < 0.001), white blood cell count (*p* < 0.001), serum chloride levels (*p* = 0.023), ACAG (*p* = 0.039), and delayed cerebral ischemia (*p* < 0.001). The AUC values for the AG, albumin, and ACAG in predicting mortality among aSAH patients were 0.606, 0.536, and 0.617, respectively. A logistic regression model, which includes the Hunt–Hess scale score, surgical options, white blood cell count, serum chloride levels, ACAG, and delayed cerebral ischemia, achieved an AUC of 0.911 for predicting mortality.

**Conclusion:**

The ACAG is an effective prognostic marker for aSAH patients. A prognostic model incorporating ACAG could help clinicians evaluate the risk of poor outcomes among aSAH patients, thereby facilitating the development of personalized therapeutic strategies.

## 1 Introduction

With a reported annual incidence of 9.1 per 100,000 people in the world, aneurysmal subarachnoid hemorrhage (aSAH) is a type of hemorrhagic stroke with mortality ranging from 8.3% to 66.7% (1, 2). The high mortality of aSAH patients is attributable to both the initial severity of hemorrhage and the subsequent complications during hospitalizations (3). Acid-base disturbance and electrolyte disorder are prevalent among aSAH patients and correlate with the prognosis of aSAH patients (4–6). Evaluating the risk and severity of these disturbances is helpful for clinicians in the risk stratification of aSAH patients and for correcting these disturbances.

As an index for diagnosing and distinguishing metabolic acidosis, the anion gap (AG) is a comprehensive and readily available marker of acid-base balance calculated based on the following formula: AG = [Na^+^ (mmol/L) + K^+^ (mmol/L)] – [Cl^−^(mmol/L) + HCO${\;}_{3}^{-}$ (mmol/L)]. The AG has been considered a marker of tissue hypoperfusion and has been confirmed to be associated with mortality due to various diseases, including acute pancreatitis, acute myocardial infarction, congestive heart failure, and sepsis (7–11). However, AG levels can be underestimated with hypoalbuminemia. To address this, the albumin-corrected anion gap (ACAG) was developed to more accurately reflect true AG levels by accounting for the influence of albumin on measuring the true level of AG. Although some studies have explored the value of AG in risk stratification for stroke patients, including those diagnosed with ischemic stroke, intracerebral hemorrhage, or intracerebral infarction (12–14), only two studies have confirmed that AG is effective in the risk stratification of subarachnoid hemorrhage patients. These studies did not evaluate the accurate prognostic value of ACAG or establish a prognostic model incorporating it (15, 16). Therefore, we conducted this study to compare the different prognostic values between AG and ACAG and to develop a prognostic model for aSAH patients using ACAG.

## 2 Materials and methods

### 2.1 Patients

**Figure 1:**
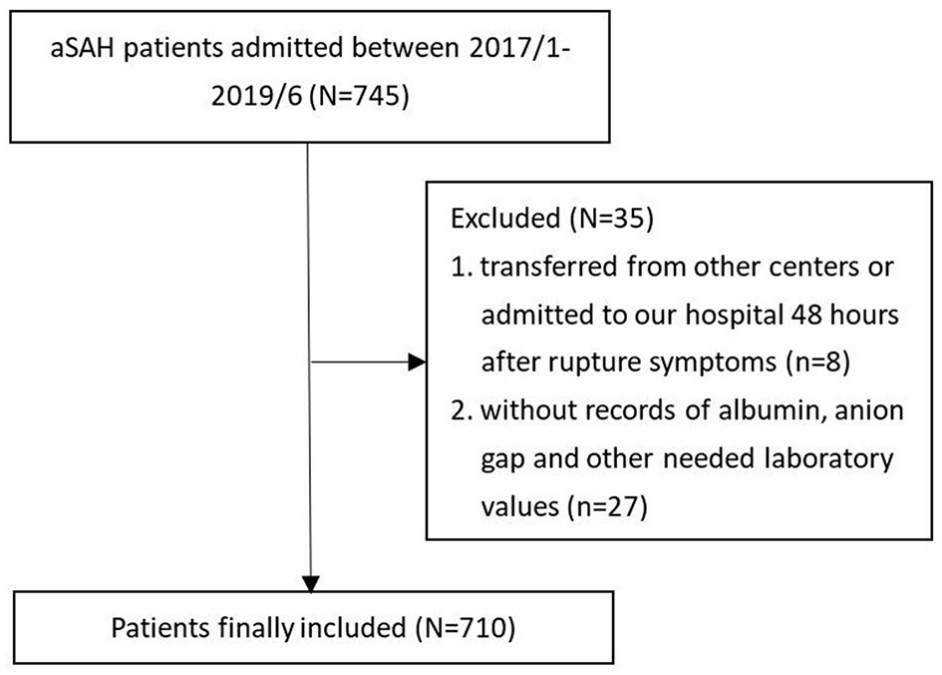
Process of aSAH patients screening.

Patients confirmed with ruptured aneurysms and those receiving treatments in the West China Hospital, Sichuan University, between 1 January 2017 and 31 June 2019 were enrolled in this observational study. Some aSAH patients were excluded from this study to ensure the reliability of the conclusions: (1) patients transferred from other medical centers or admitted to our hospital 48 h after the onset of typical symptoms for aneurysm rupture (*n* = 8) and (2) patients without a history of albumin, AG, or other needed laboratory values (*n* = 27). A total of 710 aSAH patients were eventually enrolled in the study after screening (Figure 1). This observational study was conducted with the approval of the ethical review board of West China Hospital (2021–1684) and abided by the Declaration of Helsinki.

### 2.2 Data collection

Demographical information, including age, gender, history of smoking, history of alcoholism, and history of comorbidities such as diabetes mellitus and hypertension, was recorded. Initial blood pressure on admission and conventional clinical scores specified on aSAH patients, including the World Federation Neurosurgical Society (WFNS) score, the Hunt–Hess scale score, and the modified Fisher (mFisher) scale score, were collected. Information such as the location of the aneurysm, the presence of multiple aneurysms, and the occurrence of intraventricular hemorrhage was confirmed based on radiological findings. The values of white blood cell count, hemoglobin, albumin, serum creatinine, blood urea nitrogen, serum sodium, serum potassium, serum chloride, serum calcium, and serum AG were collected from laboratory records analyzing the first blood sample after admission (within 6 h after admission). Delayed cerebral ischemia was diagnosed based on the following process: the existence of a new focal functional defect (hemiplegia, aphasia, apraxia, hemianopsia, or neglect) or a decrease in the Glasgow Coma Scale (GCS) by at least two points, excluding other causes of secondary neurological deterioration such as fever, infectious complications, hydrocephalus, seizures, respiratory failure, or electrolyte disorders. The primary outcome of this study was 30-day mortality. The follow-up using telephone interviews lasted until 1 month after the aneurysm rupture.

### 2.3 Statistical analysis

Continuous variables were shown as mean ± standard deviation (normally distributed variables) and median (interquartile range) (non-normally distributed variables). Categorical variables were shown as counts (percentages). Patients were divided into two groups (survivors and non-survivors) based on their 30-day survival. Differences in the variables between these two groups were compared using χ^2^ tests or the Fisher test (categorical variables), the Independent Student's *t-*test (normally distributed variables), and the Mann—Whitney *U*-test (non-normally distributed variables). The restricted cubic spline (RCS) was used to visually demonstrate the relationship between the ACAG level and the mortality risk of aSAH patients. The multivariate logistic regression analysis included significant factors in the univariate logistic regression analysis to explore risk factors for mortality in aSAH patients and to confirm the independent relationship between ACAG and mortality. The predictive model for mortality was developed by incorporating significant factors into the multivariate logistic regression analysis. The prognostic value of ACAG and the developed model was evaluated by calculating the area under the receiver operating characteristics curve (AUC). The Z test was used to compare the AUC between the model and other scores.

A two-sided *p* < 0.05 was defined as statistically significant. Analyses were performed using SPSS 22.0 Windows software (SPSS, Inc., Chicago, IL) and R software (Version 3.6.1).

## 3 Results

### 3.1 Baseline comparison between survivors and non-survivors

A total of 144 aSAH patients experienced 30-day mortality, including 710 patients with a mortality rate of 20.3% (Table 1). Non-survivors had older age (*p* = 0.003), higher WFNS score (*p* < 0.001), higher Hunt-Hess score (*p* < 0.001), and higher mFisher score (*p* < 0.001) than survivors. Compared to the survivors, laboratory examinations showed that white blood cell count (*p* < 0.001), serum creatinine (*p* < 0.001), blood urea nitrogen (*p* = 0.027), serum sodium (*p* = 0.002), serum chloride (*p* < 0.001), AG (*p* < 0.001), and ACAG (*p* < 0.001) were all higher in the non-survivors. In contrast, the albumin level did not show statistical significance between the survivors and the non-survivors. Compared to the survivors, the non-survivors had a higher incidence of intraventricular hemorrhage (*p* = 0.007), delayed cerebral ischemia (*p* < 0.001), and a longer length of hospital stay (*p* < 0.001).

**Table 1:** Baseline characteristics of included aSAH patients.

**Variables**	**Overall patients (*n =* 710)**	**Survivors (*n =* 566, 79.7%)**	**Non-survivors (*n =* 144, 20.3%)**	***p***
Age (year)	55 (49–65)	55 (48–64)	61 (51–69)	0.003
Male gender (%)	245 (34.5%)	196 (34.6%)	49 (34.0%)	0.892
Smoking (%)	125 (17.6%)	104 (18.4%)	21 (14.6%)	0.286
Alcoholism (%)	107 (15.1%)	89 (15.7%)	18 (12.500%)	0.334
Diabetes mellitus (%)	34 (4.8%)	29 (5.1%)	5 (3.5%)	0.407
Hypertension (%)	306 (43.1%)	251 (44.3%)	55 (38.2%)	0.183
Systolic blood pressure (mmHg)	146 (129–166)	147 (129–165)	141 (126–168)	0.491
Diastolic blood pressure (mmHg)	85 (76–95)	85 (76–95)	86 (78–95)	0.318
WFNS	2 (2–4)	2 (1–4)	4 (4–5)	< 0.001
Hunt Hess	2 (2–3)	2 (2–3)	4 (3–4)	< 0.001
mFisher	4 (2–4)	3 (2–4)	4 (3–4)	< 0.001
**Location (%)**				0.091
Anterior circulation	655 (92.3%)	527 (93.1%)	128 (88.9%)	
Posterior circulation	55 (7.7%)	39 (6.9%)	16 (11.1%)	
Multiple aneurysm (%)	68 (9.6%)	50 (8.8%)	18 (12.5%)	0.182
**Laboratory examination**				
White blood cell (10^9/*L*^)	10.34 (7.99–13.52)	9.90 (7.71–12.48)	13.20 (9.70–17.25)	< 0.001
Hemoglobin (mg/L)	125 (110–136)	125 (110–135)	125 (111–138)	0.530
Albumin (mg/dL)	3.97 (3.50–4.28)	3.99 (3.54–4.28)	3.94 (3.38–4.25)	0.182
Serum creatinine (mg/L)	57 (49–70)	56 (48–66)	68 (55–81)	< 0.001
Blood urea nitrogen (mg/L)	4.33 (3.50–5.50)	4.30 (3.40–5.40)	4.50 (3.67–5.84)	0.027
Sodium (mol/L)	139.6 (137.3–142.9)	139.5 (137.3–142.1)	140.8 (137.3–146.2)	0.002
Potassium (mol/L)	3.76 (3.50–4.08)	3.76 (3.51–4.07)	3.78 (3.43–4.11)	0.757
Chloride (mol/L)	101.9 (98.5–105.6)	101.7 (98.5–105.0)	103.5 (99.2–110.4)	< 0.001
Calcium (mol/L)	2.16 ± 0.13	2.16 ± 0.12	2.15 ± 0.15	0.621
AG (mol/L)	19.63 ± 3.55	19.32 ± 3.27	20.85 ± 4.26	< 0.001
ACAG (mol/L)	20.78 (18.65–23.00)	20.60 (18.40–22.58)	21.85 (19.35–24.68)	< 0.001
Intraventricular hemorrhage (%)	309 (43.5%)	232 (41.0%)	77 (53.5%)	0.007
Delayed cerebral ischemia (%)	117 (16.5%)	68 (12.0%)	49 (34.0%)	< 0.001
**Surgical options**				< 0.001
None	113 (15.9%)	44 (7.8%)	69 (47.9%)	
Clip	524 (73.8%)	463 (81.8%)	61 (42.4%)	
Coil	73 (10.3%)	59 (10.4%)	14 (9.722)	
Length of ICU stay (day)	3 (0–9)	3 (0–9)	4 (0–10)	0.119
Length of hospital stay (day)	12 (9–19)	13 (10–19)	7 (3–13)	< 0.001

WFNS, World Federation of Neurosurgical Societies; mFisher, modified Fisher; AG, anion gap; ACAG, albumin corrected anion gap.

### 3.2 Association between ACAG and the mortality of aSAH patients

**Figure 2:**
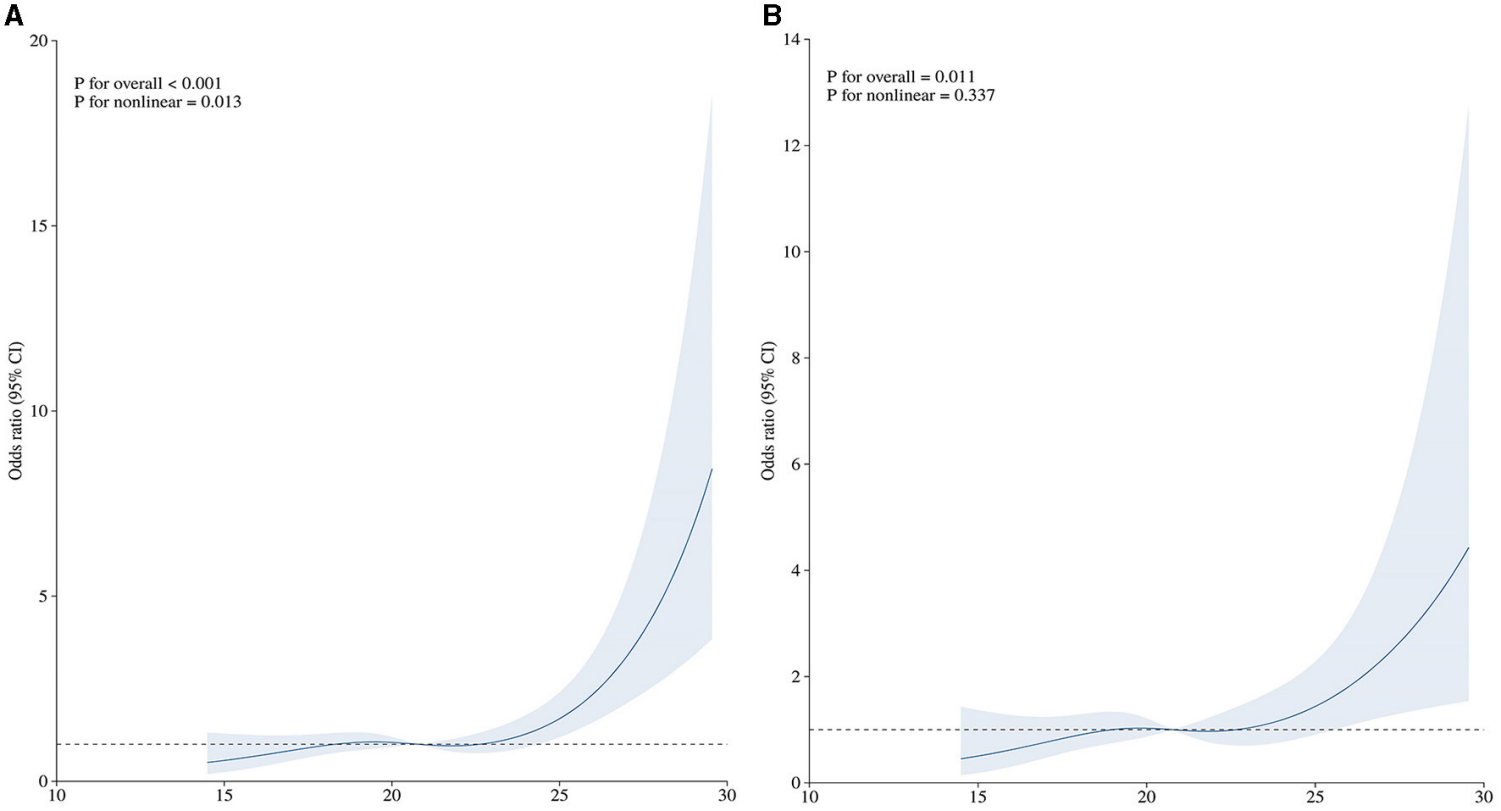
**(A)** Unadjusted association between ACAG and the mortality of aSAH patients. **(B)** Adjusted association between ACAG and the mortality of aSAH patients.

The positive relationship between the ACAG level and the mortality of aSAH patients is demonstrated using the unadjusted RCS curve shown in Figure 2A. The univariate logistic regression indicated that age (*p* = 0.001), WFNS score (*p* < 0.001), Hunt–Hess scale score (*p* < 0.001), mFisher scale score (*p* < 0.001), white blood cell count (*p* < 0.001), serum creatinine (*p* = 0.007), blood urea nitrogen (*p* = 0.046), serum sodium (*p* < 0.001), serum chloride (*p* < 0.001), ACAG (*p* < 0.001), intraventricular hemorrhage (*p* = 0.007), delayed cerebral ischemia (*p* < 0.001), surgical options (*p* < 0.001) were significantly correlated with the mortality of aSAH patients (Table 2). The multivariate logistic regression discovered that only six factors were finally and independently related to the mortality of aSAH patients after adjusting for confounding effects, including Hunt-Hess scale score (*p* = 0.006), surgical options (*p* < 0.001), white blood cells (*p* < 0.001), serum chloride (*p* = 0.023), ACAG (*p* = 0.039), and delayed cerebral ischemia (*p* < 0.001). After adjusting for the other five significant factors, the relationship between the ACAG level and the mortality of aSAH patients was still positive, as shown in Figure 2B.

**Table 2:** Univariate and multivariate logistic regression analysis of risk factors for mortality in aSAH patients.

	**Unadjusted analysis**			**Adjusted analysis**		
	**OR**	**95% CI**	***P*** **-value**	**OR**	**95% CI**	***P*** **-value**
Age	1.026	1.01–1.043	0.001	1.019	0.996–1.043	0.110
Male gender	0.974	0.662–1.432	0.892			
Smoking	0.758	0.456–1.262	0.287			
Alcoholism	0.766	0.445–1.318	0.335			
Diabetes mellitus	0.666	0.253–1.752	0.410			
Hypertension	0.776	0.533–1.128	0.184			
Systolic blood pressure	0.998	0.991–1.006	0.676			
Diastolic blood pressure	1.008	0.995–1.020	0.221			
WFNS	2.749	2.289–3.301	< 0.001	1.079	0.684–1.704	0.744
Hunt Hess	3.925	3.098–4.973	< 0.001	2.277	1.275–4.114	0.006
mFisher	1.646	1.334–2.032	< 0.001	1.340	0.923–1.967	0.129
**Location**						
Anterior circulation	1.000	Reference				
Posterior circulation	1.689	0.915–3.118	0.094			
Multiple aneurysm	1.474	0.831–2.614	0.184			
White blood cell	1.177	1.129–1.228	< 0.001	1.127	1.065–1.196	< 0.001
Hemoglobin	1.002	0.994–1.011	0.621			
Serum creatinine	1.008	1.002–1.013	0.007	1.004	0.992–1.012	0.515
Blood urea nitrogen	1.064	1.001–1.132	0.046	0.905	0.773–1.023	0.199
Sodium	1.076	1.045–1.107	< 0.001	0.984	0.896–1.078	0.725
Potassium	1.384	0.973–1.969	0.070			
Chloride	1.073	1.046–1.100	< 0.001	1.101	1.015–1.198	0.023
Calcium	0.666	0.159–2.784	0.577			
ACAG	1.158	1.097–1.222	< 0.001	1.088	1.006–1.181	0.039
Intraventricular hemorrhage	1.655	1.145–2.390	0.007	1.016	0.502–2.07	0.966
Delayed cerebral ischemia	3.777	2.463–5.794	< 0.001	4.571	2.517–8.398	< 0.001
**Surgical options**						
None	1.000	Reference				
Clip	0.084	0.053, 0.133	< 0.001	0.075	0.039–0.14	< 0.001
Coil	0.151	0.076, 0.303	< 0.001	0.122	0.046–0.298	< 0.001

WFNS, World Federation of Neurosurgical Societies; mFisher, modified Fisher; ACAG, albumin corrected anion gap.

### 3.3 Prognostic value of ACGA in aSAH patients

**Figure 3:**
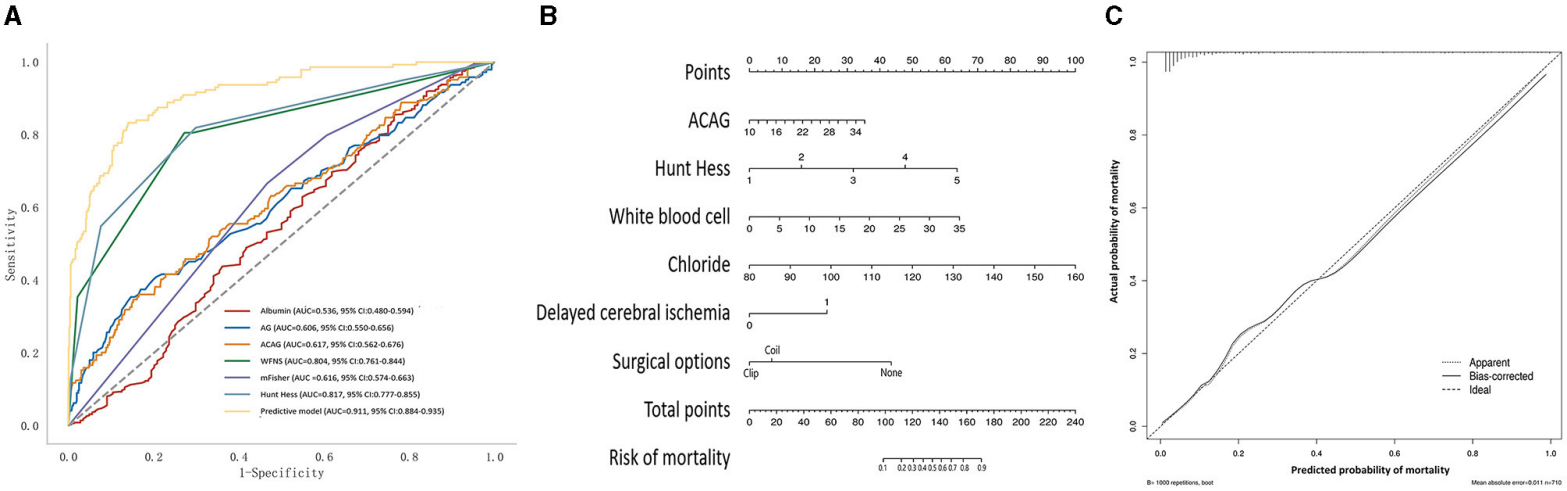
**(A)** The receiver operating characteristic curve of the prognostic model for the mortality of aSAH patients. **(B)** Nomogram of the prognostic model for the mortality of aSAH. **(C)** Calibration plot of the prognostic model for the mortality of aSAH.

The AUC values for the AG, albumin, and ACAG in predicting the mortality of aSAH patients were 0.606, 0.536, and 0.617, respectively (Figure 3A; Table 3). The AUC values for conventional scoring systems, including the WFNS, Hunt–Hess, and mFisher scale scores, were 0.804, 0.817, and 0.616. A logistic regression model composed of the Hunt–Hess scale score, mFisher scale score, white blood cell count, serum chloride levels, ACAG, and delayed cerebral ischemia achieved an AUC of 0.911, which was significantly higher than that of the WFNS score (*Z* = 6.305, *p* < 0.001), the Hunt–Hess scale score (*Z* = 6.028, *p* < 0.001), the mFisher scale score (*Z* = 12.212, *p* < 0.001), and single ACAG (*Z* = 10.466, *p* < 0.001). The nomogram and calibration plot for this model are shown in Figures 3B, C.

**Table 3:** Value of the predictive model and ACAG for predicting the mortality of aSAH patients.

**30-day mortality**						
	**AUC**	**95% CI**	**Sensitivity**	**Specificity**	**Youden index**	**Cut off**
Anion gap	0.606	0.551–0.662	0.354	0.853	0.208	22.6
Albumin	0.536	0.480–0.592	0.855	0.236	0.091	32.6
ACAG	0.617	0.563–0.670	0.347	0.848	0.195	23.8
WFNS	0.804	0.760–0.848	0.806	0.728	0.533	4
Hunt Hess	0.817	0.774–0.859	0.819	0.701	0.521	3
mFisher	0.616	0.567–0.664	0.667	0.534	0.200	4
Predictive model	0.911	0.884–0.935	0.833	0.860	0.694	0.208

AUC, area under the receiver operating characteristic curve; CI, confidence interval; WFNS, World Federation of Neurosurgical Societies; ACAG, albumin corrected anion gap.

The predictive model was composed of Hunt Hess, surgical options, white blood cell, serum chloride, ACAG, delayed cerebral ischemia.

## 4 Discussion

The prevalence of delayed cerebral ischemia and mortality was 16.5% and 20.3%, which was similar to the recently reported incidence (17–20). The high mortality rate makes risk evaluation crucial. As a readily available marker of acid-base balance, AG may be beneficial for the risk evaluation of prognoses among aSAH patients. The results of this study showed that, among aSAH patients, the non-survivors had higher levels of AG and ACAG than the survivors. The accuracy of ACAG in predicting the mortality of aSAH patients was higher than that of the original AG. As a marker identifying metabolic acidosis, the AG increased due to the overproduction of organic acids or the reduced renal excretion of anion (21). It is worth noting that aSAH leads to acidosis through multiple mechanisms, including hypoxia of cerebral tissue, accumulation of acidic metabolites, and other electrolyte disorders such as hypokalemia and hypocalcemia (4–6). Additionally, impaired renal function, which is commonly observed among aSAH patients, causes the accumulation of acidic metabolites (22–24). Therefore, the increased AG after aSAH may comprehensively reflect the abnormal metabolic state and renal dysfunction and thus contribute to the higher risk of mortality. Additionally, it could be deduced that elevated serum sodium levels cause an increase in AG based on the following formula: AG = [Na^+^ (mmol/L) + K^+^ (mmol/L)] – [Cl^−^(mmol/L) + HCO${\;}_{3}^{-}$(25). The detrimental effect of hypernatremia on the prognosis of aSAH patients has been proven by several studies (6, 26–28). Finally, high serum AG levels have been found to be related to a high level of inflammatory biomarkers, including white blood cells and C-reactive protein (29). The inflammatory response after aSAH plays an important role in the pathophysiological process of brain injury and extracranial organ dysfunction (30–33).

Although many studies have explored the prognostic value of AG in various diseases, including acute pancreatitis, acute myocardial infarction, congestive heart failure, and sepsis, they have not compared the distinct impacts of single AG vs. ACAG (7–11). AG levels are often underestimated in cases of hypoalbuminemia, which commonly occurs during hospitalizations of aSAH patients (34). This underestimation may obscure the actual effect of acidosis on the prognosis of aSAH patients. Moreover, two recent studies that used AG for risk stratification in subarachnoid hemorrhage patients did not explore the supplementary effect of albumin correction or develop a prognostic model using ACAG (15, 16). The prognostic value of the ACAG was higher than that of the single AG in our study. When the measured AG is at the same level, a lower albumin level indicates a higher ACAG. This finding indicates that a high ACAG also partially reflects the effect of hypoalbuminemia on the prognosis of aSAH patients. Previous studies showed that hypoalbuminemia is associated with poor prognosis and complications after aSAH, including pneumonia, cerebral vasospasm, and delayed cerebral ischemia (35–38).

In addition to the AG, blood gas analysis could also be used to evaluate acid-base disturbances. The result of the blood gas analysis could be disturbed by compensatory respiratory alkalosis. Compared with the blood gas analysis needing arterial puncture, the AG is less expensive and more easily available in situations with limited resources (39). After being corrected using the albumin, the ACAG is useful for clinicians when evaluating the risk of poor prognosis among aSAH patients with relatively fewer resources.

Some limitations of this study should be noted. First, only the ACAG at admission was recorded, and any changes in ACAG were not tracked over time, so we could not evaluate the prognostic value of ACAG at different time points or ACAG changes among aSAH patients. Second, the record of blood lactate levels in our patient cohort was not complete, limiting our ability to compare the different values of lactate and ACAG. Third, only the survival outcome (30-day mortality) was analyzed; however, no other outcomes, including functional status and long-term cognitive status, were analyzed. The association between functional outcome and ACAG has been analyzed in our preliminary study, but we found that ACAG was not effective in predicting the functional outcome and thus did not include these results. Fourth, some confounders influencing the level of AG were not analyzed, including renal excretory function, the intravenous infusion volume, and the use of dehydration drugs. Fifth, some aneurysm-related morphological information, such as aneurysm size and aneurysm shape, was not collected. Our developed model may be improved after adding these variables. Finally, this study was conducted using the data from a single medical center in the southwestern region of China, highlighting the need for future studies in other regions and with larger sample sizes to validate our findings.

## 5 Conclusion

The ACAG is a prognostic marker for aSAH patients and could guide clinicians in correcting acid-base imbalances. A prognostic model incorporating ACAG could help clinicians evaluate the risk of poor outcomes among aSAH patients, thereby facilitating the development of personalized therapeutic strategies.

## Data availability statement

The raw data supporting the conclusions of this article will be made available by the authors, without undue reservation.

## Ethics statement

The studies involving humans were approved by the Ethics Committee of West China Hospital. The studies were conducted in accordance with the local legislation and institutional requirements. The participants provided their written informed consent to participate in this study.

## Author contributions

RW: Conceptualization, Data curation, Formal analysis, Writing – original draft. JR: Data curation, Formal analysis, Writing – original draft. MH: Writing – review & editing. JX: Writing – review & editing.
